# Supplementary material for: Content-rich biological network constructed by mining PubMed abstracts
Source: BMC Bioinformatics. 2004 Oct 8;5:147. doi: 10.1186/1471-2105-5-147 (PMC528731; doi:10.1186/1471-2105-5-147)
Supplement: Additional File 2 — The original results of the above study (non-essential files are deleted to keep the file size under the limit set by BMC bioinformatics). [file 1471-2105-5-147-S2.bz2 › chilibotAdditionalFile2/dip05/113ID8645696E457/html/ATPA_ATPH.html]

 


 **ATPA** and **ATPH** 
  
Found 15 abstracts in PubMed, retrieved 05.  
 

 What does Google say? 
 PDF only 
| .edu only 

---

**Interactive relationship** (e.g. stimulation, inhibition, etc)

**Neutral relationship**- This paper describes the  **atpA**  gene cluster in the C. reinhardtii chloroplast genome, which includes the  **atpA** , psbI, cemA, and  **atpH**  genes, encoding the alpha subunit of the coupling factor 1 CF1 ATP synthase, a small photosystem II polypeptide, a chloroplast envelope membrane protein, and subunit III  [ **ATPH** ]  of the CF0 ATP synthase, respectively.  Ref: 9625716 Plant Physiol, 1998

**Non-interactive relationship** (e.g. studied together, co-existance, homology, etc.)

- We show that promoters precede the  **atpA** , psbI, and  **atpH**  genes, but not the cemA gene,.  Ref: 9625716 Plant Physiol, 1998
- No genes for photosynthetic functions have been found, except for sequences encoding six subunits of the ATP synthase  **atpA** , atpB, atpE, atpF,  **atpH** , and atpI.  Ref: 12111556 Mol Genet Genomics, 2002
- The C. thermoaceticum atp operon contained nine genes arranged in the order atpI i, atpB a, atpE c, atpF b,  **atpH**  delta,  **atpA**  alpha, atpG gamma, atpD beta, and atpC epsilon.  Ref: 9171425 J Bacteriol, 1997
- Eight genes atpI, atpB, atpE 1, atpE 2, atpE 3, atpF,  **atpH** , and  **atpA**  upstream of and contiguous with the previously described genes atpG, atpD, and atpC were cloned from chromosomal DNA of Acetobacterium woodii.  Ref: 10567365 J Biol Chem, 1999
